# Supplementary figures and images for: Genetically Modified Heat Shock Protein90s and Polyamine Oxidases in Arabidopsis Reveal Their Interaction under Heat Stress Affecting Polyamine Acetylation, Oxidation and Homeostasis of Reactive Oxygen Species
Source: Plants (Basel). 2019 Sep 3;8(9):323. doi: 10.3390/plants8090323 (PMC6783977; doi:10.3390/plants8090323)

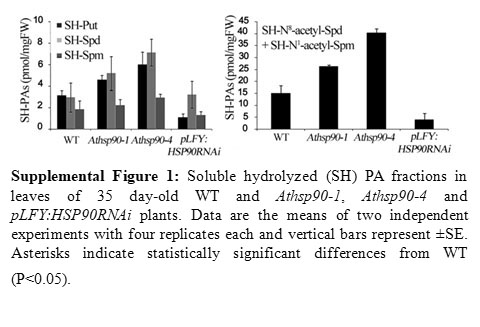

Supplement: Supplementary file 1 [file plants-08-00323-s001.zip › Figure S1.jpg]

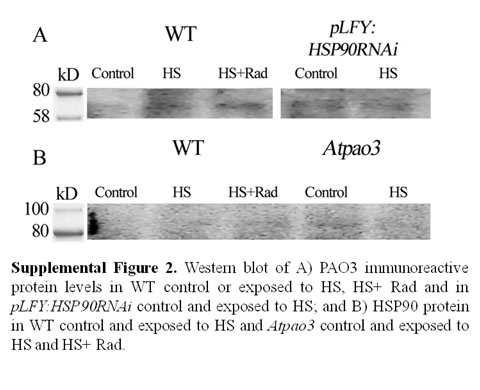

Supplement: Supplementary file 1 [file plants-08-00323-s001.zip › Figure S2.jpg]

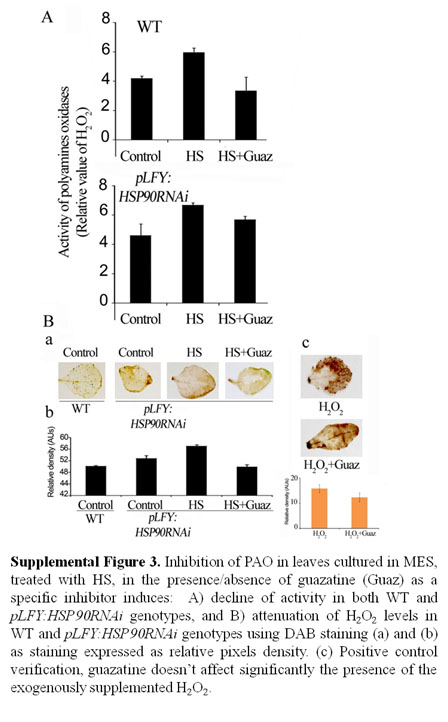

Supplement: Supplementary file 1 [file plants-08-00323-s001.zip › Figure S3.jpg]

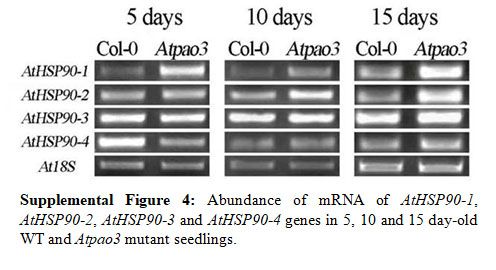

Supplement: Supplementary file 1 [file plants-08-00323-s001.zip › Figure S4.jpg]

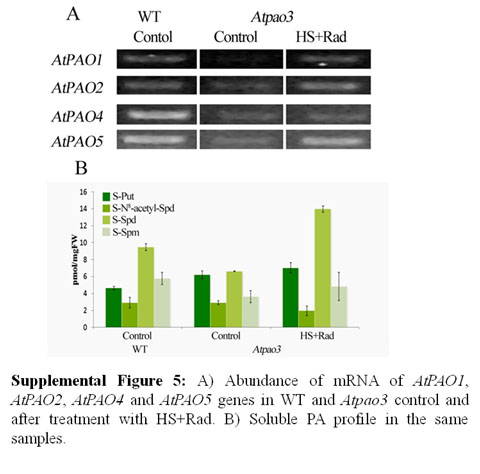

Supplement: Supplementary file 1 [file plants-08-00323-s001.zip › Figure S5.jpg]

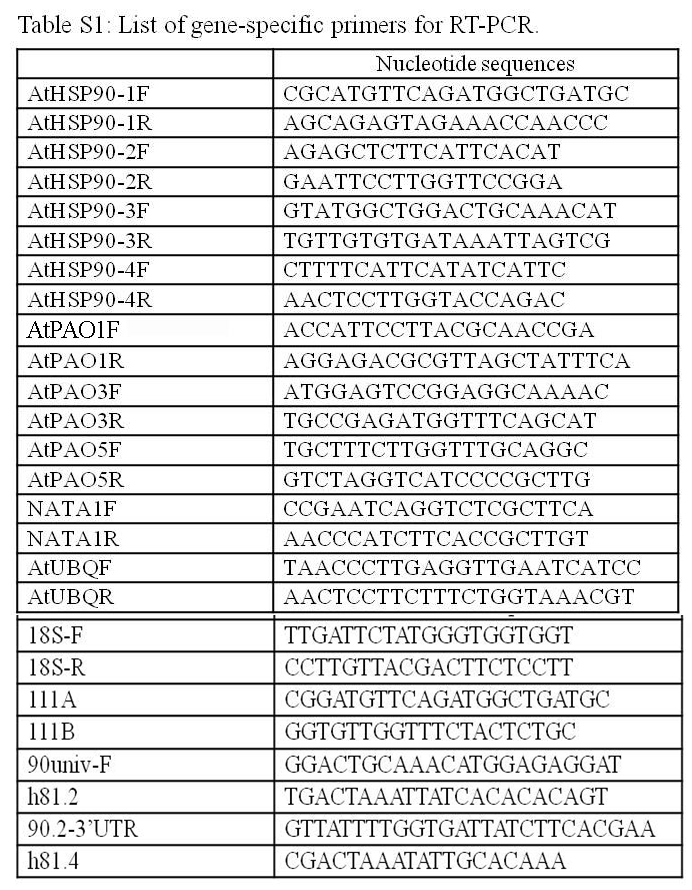

Supplement: Supplementary file 1 [file plants-08-00323-s001.zip › Table S1.png]
